# Procalcitonin-Based Antibiotic Use for Neonatal Early-Onset Bacterial Infections: Pre- and Post-Intervention Clinical Study

**DOI:** 10.3390/antibiotics12091426

**Published:** 2023-09-09

**Authors:** Hidetoshi Go, Nobuhiko Nagano, Yuki Sato, Daichi Katayama, Koichiro Hara, Takuya Akimoto, Takayuki Imaizumi, Ryoji Aoki, Midori Hijikata, Ayako Seimiya, Aya Okahashi, Ichiro Morioka

**Affiliations:** 1 Department of Pediatrics and Child Health, Nihon University School of Medicine, Tokyo 1738610, Japan; go.hidetoshi@nihon-u.ac.jp (H.G.); nagano.nobuhiko@nihon-u.ac.jp (N.N.); sato.yuki@nihon-u.ac.jp (Y.S.); katayama.daichi@nihon-u.ac.jp (D.K.); hara.koichiro@nihon-u.ac.jp (K.H.); akimoto.takuya@nihon-u.ac.jp (T.A.); imaizumi.takayuki@nihon-u.ac.jp (T.I.); ryoji_kamo@yahoo.co.jp (R.A.); hijikata.midori@nihon-u.ac.jp (M.H.); seimiya.ayako78@nihon-u.ac.jp (A.S.); okahashi.aya@nihon-u.ac.jp (A.O.); 2 Department of Radiology, Nihon University School of Medicine, Tokyo 1738610, Japan

**Keywords:** antibiotics, antibiotic resistance, appropriate use of antibiotics, days of antibiotic therapy, early-onset bacterial infection, procalcitonin

## Abstract

We previously reported the 95th percentile cutoff value of the serum procalcitonin (PCT) reference curve for diagnosing early-onset bacterial infection. We aimed to verify the effectivity of these novel diagnostic criteria by comparing antibiotic use and incidence of early-onset bacterial infection between pre- and post-introduction periods. We included newborns admitted to our neonatal intensive care unit who underwent blood tests within 72 h after birth between 2018 and 2022. The neonates were divided into the pre-intervention (admitted before the introduction, *n* = 737) or post-intervention (admitted after the introduction, *n* = 686) group. The days of antibiotics therapy (DOT) per 1000 patient days up to 6 days after birth, percentage of antibiotic use, and incidence of early-onset bacterial infection were compared between the groups. The post-intervention group had significantly lower DOT per 1000 patient days (82.0 days vs. 211.3 days, *p* < 0.01) and percentage of newborns receiving antibiotics compared with the pre-intervention group (79 (12%) vs. 280 (38%), respectively, *p* < 0.01). The incidence of early-onset bacterial infections did not differ between the groups (2% each, *p* = 0.99). In conclusion, our diagnostic criteria using the 95th percentile cutoff value of the serum PCT reference curve for early-onset bacterial infection were proven safe and effective, promoting appropriate use of antibiotics.

## 1. Introduction

The increasing prevalence of drug-resistant bacteria populations, also known as antibiotic resistant bacteria (AMR), has become a global area of concern. Annually, approximately 700,000 individuals worldwide die from infections caused by AMR, and by 2050, the emergence of new AMR is projected to lead to an annual death of 10 million people worldwide unless proactive actions are taken to mitigate its spread. The worldwide economic cost of AMR by 2050 is estimated to reach as high as USD 100 trillion, underscoring the need to address this concern from an economic perspective [1]. In addition, a decreasing trend has been observed in the development of new antibiotic agents [2]. Thus, there is a need to curb the development of drug resistance in bacteria and preserve the availability of effective antibiotics for future generations.

AMR control is also an issue of concern in neonatal intensive care units (NICUs). Excessive or careless use of antibiotics drugs, particularly third-generation cephalosporins and carbapenems, can lead to the persistence and spread of multidrug-resistant Gram-negative rod *bacilli* in addition to invasive *Candida* infections [3]. The use of these drugs is known to lead to *Candida* colonization in the gastrointestinal tract of neonates, especially extremely preterm neonates. *Candida* colonization can be a risk factor for invasive candidiasis. AMR can be mitigated by avoiding the use of third-generation cephalosporins, which, in turn, can reduce substrate-specific extended-spectrum β-lactamase (ESBL)-producing Gram-negative bacterial infections [4,5]. Bacteremia caused by multidrug-resistant Gram-negative rods accounts for 18.6% of the Gram-negative rod bacteremia in NICUs, which is particularly prevalent in neonates who have undergone treatment with broad-spectrum antibiotic agents and have underlying renal disease, highlighting the urgent need for remedial measures [6].

Prolonged early antibiotic use in markedly low-birth-weight infants increases the risk of necrotizing enterocolitis and death [7]. The development and increased use of broad-spectrum antibiotics has been reported to increase the emergence of AMR, with a 200–800% increase in mortality from AMR-associated bacteremia [6]. Moreover, antibiotic use during the neonatal period can potentially increase the risk of allergy, asthma, and obesity in the future [8], and the use of antibiotics in patients aged < 2 years is a risk for allergic disease by age 5 [9]. Antibiotic use in childhood may also increase the risk of developing Crohn’s disease and juvenile idiopathic arthritis [10,11]. Thus, appropriate use of antibiotic drugs has the potential to not only improve outcomes during NICU admission but also mitigate various long-term disease risks.

However, there can be difficulty in recognizing early symptoms in neonates, who often receive antibiotic therapy even though they do not have an infectious disease, because symptoms of neonatal infection are nonspecific. This is due to the consequential serious sequelae and substantial mortality rate associated with neonatal bacterial infections, underscoring the importance of timely diagnosis and treatment [12,13,14,15].

Currently, various biomarkers have been investigated for the timely diagnosis of early-onset neonatal bacterial infections [16,17,18,19]. In this study, we focused on procalcitonin (PCT), which is a precursor of calcitonin. It is produced and secreted by the thyroid C cells in the normal physiological state. However, proinflammatory cytokines triggered by bacterial organisms and toxins stimulate the liver to generate PCT. Notably, PCT levels are not elevated in response to viral infections due to the inhibitory effect of the interferon-gamma, which is produced during viral infections [20]. During the early postnatal period, PCT levels exhibit physiological fluctuation, with distinct patterns observed in preterm and term infants. Moreover, increased PCT levels are observed not only during infection but also during hypoxemia, the onset of respiratory disorders, asphyxia, intracranial hemorrhage, and circulatory abnormalities [21,22,23,24,25,26]. To address these physiological variations, a reference curve for PCT values during the early postnatal period has been proposed [21,22,23,24].

Previous studies on the association between PCT and neonatal sepsis have reported a sensitivity of 78% and a specificity of 50% at a PCT cutoff value of 0.5 ng/mL; sensitivity of 58% and specificity of 83% at a cutoff value of 1 ng/mL; sensitivity of 83% and specificity of 61% at a cutoff value of 2 ng/mL; and a sensitivity of 68% and a specificity of 67% at a cutoff value of 5.75 ng/mL [27,28,29,30]. Collectively, these findings indicate that relying solely on sensitivity and specificity is insufficient for diagnosing neonatal sepsis.

Therefore, we proposed a novel diagnostic criterion involving the 95th percentile value derived from the PCT value reference curve. Our findings demonstrated the application of this criterion for diagnosing neonatal early-onset bacterial infection in both preterm and term infants [31]. Specifically, the 95th percentile value of the PCT value reference curve is used alone in preterm infants, while it is used in combination with C-reactive protein (CRP), white blood cell (WBC) count, and immunoglobulin M (IgM) values in term infants. This new diagnostic criterion exhibited excellent diagnostic accuracy for early-onset bacterial infection in neonates, with sensitivity, specificity, and a Youden index of 1.00 [27,32]. Therefore, our hypothesis was that the introduction of PCT-based criteria can reduce the unnecessary use of antibiotics in NICUs. In this study, we aimed to determine whether the newly introduced diagnostic criteria would effectively reduce the frequency of antibiotic use among infants in the NICU.

## 2. Results

### 2.1. Study Population and Baseline Characteristics

**Figure 1:**
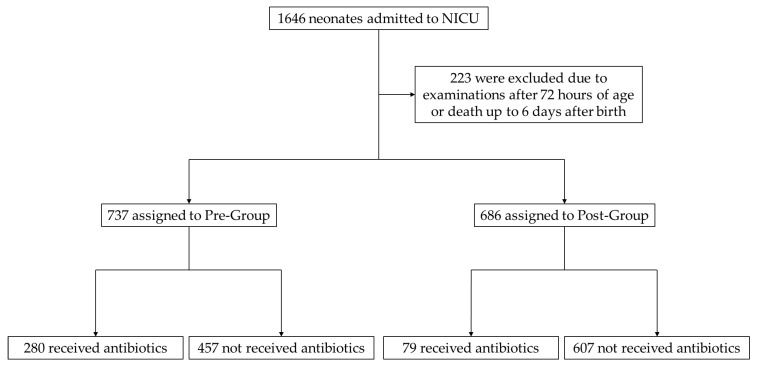
Flowchart of the participants. NICU, neonatal intensive care unit.

During the study period, 1646 neonates were hospitalized; 217 were excluded as they had not been examined within 72 h of birth, and 6 died before 6 days of age. Overall, 737 neonates were included in a pre-intervention group (Pre-Group) and 686 in a post-intervention group (Post-Group) (Figure 1). There were no significant differences in maternal background factors between the two groups, such as gestational diabetes (GDM), hypertensive disorder of pregnancy (HDP), *Streptococcus agalactiae* (GBS) colonization, clinical chorioamnionitis, or intrapartum antibiotic therapy. The Pre-Group exhibited a significantly higher proportion of premature rupture of the membranes (PROM) than the Post-Group. There were no significant differences in neonatal background between the two groups, such as gestational age at birth, birth weight, Apgar score, respiratory disorder, severe neonatal asphyxia, patent ductus arteriosus (PDA) requiring treatment, early-onset bacterial infection, death, death due to infection, and use of catecholamines, steroids, and gamma globulin. In the Pre-Group, neonatal transport and surgical conditions were significantly more common than those in the Post-Group. Age at examination was significantly younger and the CRP levels were significantly higher in the Pre-Group than the Post-Group. Meanwhile, the WBC and IgM did not differ significantly between the two groups (Table 1).

Table 2 and Table 3 show the details of early-onset bacterial infection in the Pre- and Post-Groups, respectively. In the Pre-Group, GBS was the most common pathogen (seven patients), and culture-proven sepsis was diagnosed in three patients. In the Post-Group, GBS and *Escherichia coli* were detected in four patients and three patients, respectively, and culture-proven sepsis was diagnosed in one patient. In the Pre-Group, most of the cases of early-onset bacterial infection were vaginal deliveries, and the majority were term infants. In the Post-Group, many of the patients were born by cesarean section due to the preterm deliveries.

### 2.2. Antibiotic Therapy

**Figure 2:**
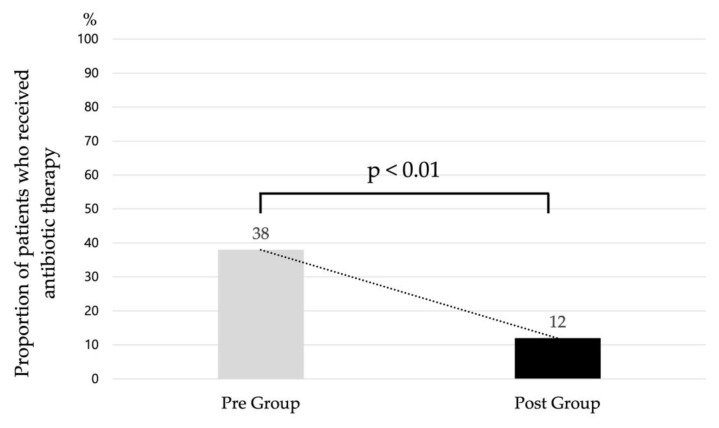
Change in the proportion of patients who received antibiotic therapy between the Pre- and Post-Groups.

The number of patients who received antibiotics decreased significantly from 280 (38%) in the Pre-Group to 79 (12%) in the Post-Group (*p* < 0.01, Figure 2). Days of antibiotics therapy (DOT) per 1000 patient days significantly decreased from 211.3 in the Pre-Group to 82.0 in the Post-Group (*p* < 0.01). Moreover, the total, ampicillin, and amikacin DOT significantly decreased in the Post-Group. Cephalosporin, penicillin, meropenem, tazobactam/piperacillin, and vancomycin DOT did not differ significantly between the two groups (Table 4).

Neonates who received antibiotics were 280 in the Pre-Group and 79 in the Post-Group. We set the cutoff value at 3 days because the culture results are confirmed in approximately 3 days in our hospital. Regarding the duration of antibiotic use, the Post-Group had significantly fewer patients with 1–3 days and more patients with 4–7 days than the Pre-Group (*p* < 0.01, Table 5).

### 2.3. Propensity Score Analysis, Multiple Regression Analysis, and Logistic Regression Analysis

**Figure 3:**
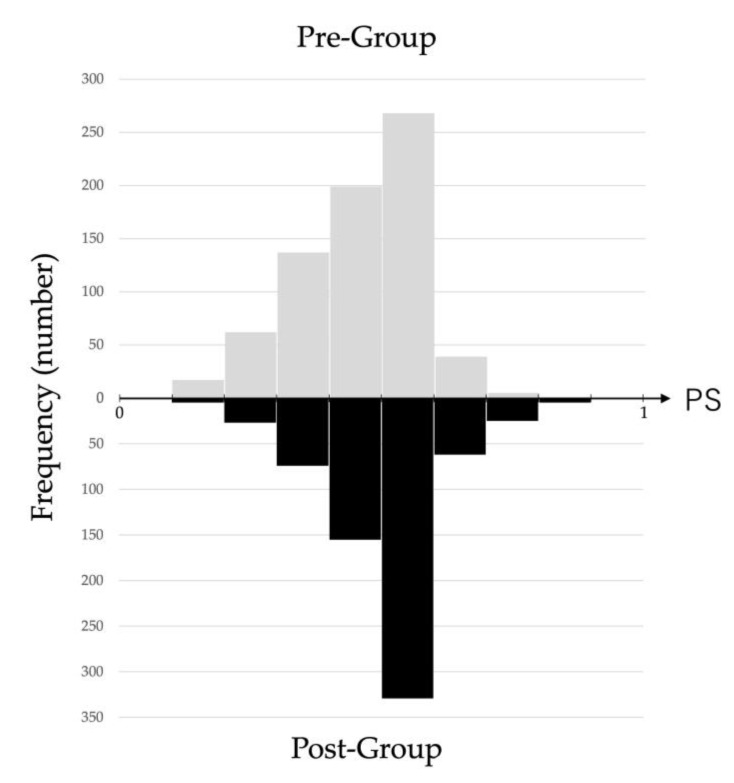
The histograms of Pre-Group and Post-Group. Based on the frequency of trends obtained from the PS, the histograms of Pre-Group and Post-Group are shown in the upper and lower rows, respectively. PS, propensity score.

Figure 3 shows the histograms of the Pre-Group and Post-Group obtained from propensity scores (PS). Table 6 shows the results of multiple regression analysis with intervention and PS as explanatory variables and DOT as outcome. Significant differences were found between the Pre-Group and Post-Group, even when adjusted for PS. The variance inflation factor for the explanatory variables, intervention, and PS was 1.06, indicating no multicollinearity. Table 7 shows the results of logistic regression analysis with intervention, PS, and the use of antibiotic as explanatory variables. The results also showed a significant difference between the Pre-Group and Post-Group, even after adjusting for PS.

## 3. Discussion

With the increasing prevalence of AMR and the current lack of progress in the development of new antibiotics, preventing unnecessary use of antibiotics is crucial in reducing AMR and improve the survival prognosis of neonates. Thus, the findings of the present study highlight that the new PCT diagnostic criteria can facilitate appropriate use of antibiotic therapies.

Although maternal characteristics showed a significantly higher rate of PROM in the Pre-Group, there were no significant differences between the two groups in the intrapartum antibiotic therapy or in the occurrence of early-onset bacterial infections. Therefore, administration of antibiotics to neonates for PROM is considered not essential. In the Pre-Group, the proportion of neonatal transports was significantly higher than the Post-Group. In the Pre-Group, the percentage of antibiotic use among transported infants was 44%, which influenced the use of antibiotics. Conversely, in the Post-Group, antibiotics were used in 14% of the cases. Among the cases of suspected neonatal infections in which the neonate was transported, antibiotic use was 95% in the Pre-Group and 12.5% in the Post-Group, leading to a significant decrease in antibiotic use. Although CRP levels were significantly greater in the Pre-Group than the Post-Group, the reason for high CRP level is considered to be physiological elevation [22] because no significant difference was observed in the diagnosis of early-onset bacterial infections between the two groups.

GBS was the most common pathogen detected among those diagnosed with early-onset bacterial infection in both groups, which is similar to the previous reports of early-onset sepsis [33]. GBS was common in term infants, and *Escherichia coli* was common in preterm infants, which was consistent with previous reports [34,35].

Our new PCT diagnostic criteria for early-onset bacterial infections significantly reduced the number of patients that used antibiotics, and no patients died of infection, suggesting that antibiotic use could be safely reduced. The findings revealed that antibiotic use was unnecessary in the Pre-Group. In the Post-Group, antibiotics were prescribed to 65 patients despite the patients not contracting bacterial infections. These included 21 patients with respiratory disorder, 15 patients with extremely preterm birth, 8 patients with surgical conditions, and 7 patients exhibiting severe asphyxia. The use of antibiotic drugs was necessary in infants with surgical conditions as the postoperative administration cannot be avoided. A future issue to address will be reducing the unnecessary use of antibiotics in extremely preterm infants and those with respiratory disorders.

DOT decreased significantly in the Post-Group. Specifically, there was a significant decline in the use of ampicillin and amikacin, which are often administered as initial antibiotic treatments in Japan. This decrease was attributed to a reduction in unnecessary prophylactic antibiotic administration. There were no significant differences in the use of cephalosporin, penicillin, meropenem, and tazobactam/piperacillin between the Pre- and Post-Groups. In the Post-Group, cephalosporin was prescribed to eleven patients, with four cases involving bacterial infection and five cases involving surgical conditions. Reducing such use was considered difficult due to the essential nature of the use. Third-generation cephalosporin was not used in the Post-Group except in cases of bacterial infection, a favorable result from the perspective of AMR suppression. Penicillin was administered in cases of congenital syphilis, which was deemed necessary. The broad-spectrum antibiotic meropenem and tazobactam/piperacillin were prescribed in cases of septic shock, and their use was considered unavoidable. There was no occurrence of methicillin-resistant *Staphylococcus aureus* infections during the study period, and vancomycin was not prescribed.

In relation to patients who received antibiotic therapy, the duration of antibiotic use was comparatively shorter in the Pre-Group and longer in the Post-Group. In the Pre-Group, several patients received prophylactic antibiotic treatment due to elevated CRP levels, preterm births, and respiratory disorders. Many of these patients completed the course of antibiotic therapy within 3 days after confirming the negative culture results upon admission. Consequently, a number of unnecessary prophylactic antibiotic administrations occurred in the Pre-Group, resulting in a substantial number of patients with a short antibiotic usage period. Regression analysis adjusted for bias of background factors in Table 2 using PS also showed significant differences in DOT and antibiotic use. Therefore, the diagnostic criteria were considered useful.

In the present study, the percentage of antibiotic use decreased significantly in the Post-Group because of unnecessary antibiotic administration in the Pre-Group. Notably, the antibiotic use in the Pre-Group did not surpass the reported usage in previous studies [36,37,38,39,40]. Therefore, the frequency and amount of antibiotic use in our NICU was not considered to be particularly high, even in the Pre-Group.

The administration of antibiotics to neonates disrupts the intestinal microbiota [41], which is potentially associated with the development of allergic diseases, juvenile idiopathic arthritis, and autism spectrum disorders in the future [9,10,11,42]. Disrupting the development of the microbiome contributes to the increased prevalence of AMR. The reduction in antibiotic use not only reduces long-term disease risk but also deters the emergence of AMR.

Various efforts are underway to reduce inappropriate use of antibiotics in the NICU. The review paper has categorized methods of reducing antibiotic use as those focusing on (1) initiating antibiotic therapy, (2) reducing the duration of antibiotic treatment, and (3) introducing programs to support appropriate antibiotic use [43]. The present study found that the implementation of focusing on initiating antibiotic therapy reduced the use of antibiotics. In the future, further reduction in antibiotic use may be expected by shortening the duration of antibiotic treatment and introducing programs to support appropriate antibiotic use. The Post-Group in this present study showed a reduction in pharmacoeconomic costs for the use of antibiotics compared to the Pre-Group.

This study is subject to several limitations. First, because this was a single-center study, there were no mortalities attributable to infection in either the Pre- or Post-Group, making it difficult to determine the occurrence of treatment failure. Secondly, the criteria for termination of antibiotic therapy were determined by the attending physician. Clearly defined criteria for termination of antibiotic therapy could further reduce DOT and decrease the number of neonates who undergo unnecessary antibiotic treatment.

## 4. Materials and Methods

### 4.1. Study Design and Patients

Neonates admitted to the NICU of the Nihon University Itabashi Hospital between 6 March 2018 and 5 March 2022 were included. Some neonates were born in other hospitals and transported to our NICU when neonates showed unusual symptoms or signs after birth that may have required medical treatment. The neonates were divided into two groups: the Pre-Group admitted between 6 March 2018 and 5 March 2020 and the Post-Group admitted between 6 March 2020 and 5 March 2022. Patients in both groups who did not undergo blood tests within 72 h of birth or who died before day 6 were excluded. Blood tests were performed in all patients to evaluate CRP, WBC count, and IgM levels. Meanwhile, the PCT levels were additionally evaluated in the post-intervention group. Furthermore, all patients underwent blood, nasal, stool, urine, intragastric amniotic fluid, and spinal fluid cultures, as needed. This study was approved by the Institutional Review Board of Nihon University Itabashi Hospital (RK-190910-8 and RK-230314-10). This study was also registered with the Clinical Trials Registry in Japan (UMIN: R000059333). Patient consent was waived because this study contains no identifiable private information. We also posted an opt-out notification on the hospital homepage “http://www.med.nihon-u.ac.jp/hospital/itabashi/cr/index.html (accessed on 1 September 2023)”.

### 4.2. Decision to Administer Antibiotics

**Figure 4:**
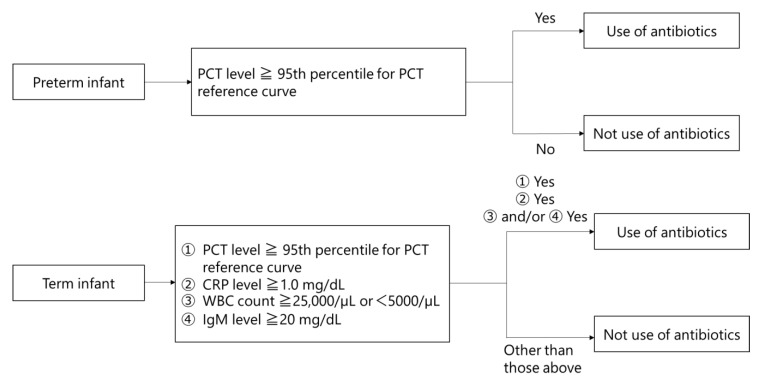
Protocol for use of antibiotics in preterm and term infants. CRP, C-reactive protein; IgM, immunoglobulin M; PCT, procalcitonin; WBC, white blood cell.

**Figure 5:**
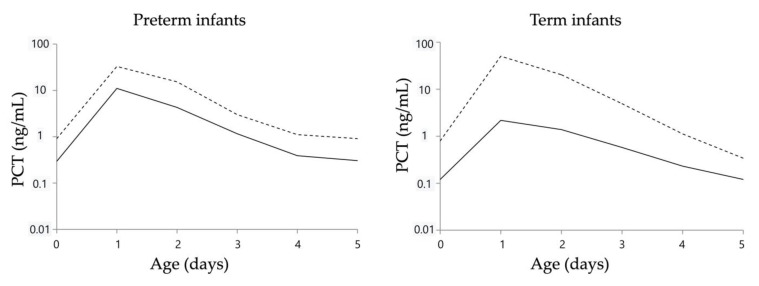
Serum PCT reference curves for preterm and term infants. These figures are cited from reference [21]. Solid and dotted lines indicate the 50th and 95th percentile lines, respectively. PCT, procalcitonin.

The decision to administer an antibiotic in the Post-Group was made using the PCT value reference curve for preterm infants and a combination of the PCT value reference curve and CRP, WBC count, and IgM values for term infants. Specifically, in preterm infants, an antibiotic was administered if the PCT value exceeded the 95th percentile of the PCT value reference curve. In term infants, an antibiotic was administered if the PCT value exceeded the 95th percentile of the PCT value reference curve in addition to a WBC count ≥25,000/µL or <5000/µL or IgM ≥ 20 mg/dL and a CRP value ≥ 1.0 mg/dL (Figure 4) [31]. The PCT value reference curve was the curve with the highest diagnostic accuracy in previous reports (Figure 5) [21]. In the Pre-Group, this was performed at the discretion of the attending neonatologists.

### 4.3. Diagnosis of Bacterial Infection

A diagnosis of neonatal bacterial infection was reached when pathogens were detected in culture tests within 6 days after birth. Culture-proven sepsis was diagnosed when pathogens were detected in blood cultures. A diagnosis of clinical sepsis was made when pathogens were detected in nasopharyngeal swab, stool, urine, intragastric amniotic fluid, or spinal fluid cultures in addition to three or more of the following findings: respiratory, circulatory, and neurologic symptoms; abnormal body temperature and skin color; or delayed capillary filling time [44,45,46].

### 4.4. Methods of Measuring Each Biomarker and Culture

Serum PCT, CRP, and IgM levels were measured using Lumipulse Presto Brahms PCT kits (FUJIREBIO Inc., Tokyo, Japan), an LZ Test EIKEN CRP-HG (Eiken Chemical Co., Ltd., Tokyo, Japan), and an N-assay TIA IgM-SH Nittobo (Nittobo Medical Co., Ltd., Tokyo, Japan), respectively, as recommended by the manufacturers. WBC was measured using an automated hematology analyzer (XN-9100; Sysmex Co., Ltd., Kobe, Japan).

For cultures, blood, nasopharyngeal swab, and stool samples were collected using BD BACTEC™ Peds Plus™ Media (Becton, Dickinson and Company, Franklin Lakes, NJ, USA), Transystem™ 116C (COPAN Diagnostics Inc., Murrieta, CA, USA), and Transystem™ 108C (COPAN Diagnostics Inc., Murrieta, CA, USA), respectively. Urine, gastric aspirate, and cerebrospinal fluid samples were collected in sterile spitz (Eiken Chemical Co., Ltd., Tokyo, Japan). Cultures were performed using blood and chocolate agar to identify the organisms. Positive blood culture results were detected using the BD BACTEC™ FX System (Becton, Dickinson and Company, Franklin Lakes, NJ, USA), as recommended by the manufacturer.

### 4.5. Study Methods

#### 4.5.1. Clinical Characteristics of Each Group and Causative Organisms of Early-Onset Neonatal Bacterial Infections

Maternal background factors in the Pre- and Post-Groups included pregnancy complications (GDM [47,48,49] and HDP [49,50]), vaginal culture positive for GBS, clinical chorioamnionitis [51,52], PROM [53]), delivery mode, and precipitate delivery, and the number of patients who received intrapartum antibiotic drug therapy were compared. Additionally, a comparison of the neonatal background factors including gestation age at birth; birth weight; Apgar scores at 1 and 5 min; transport from another hospital; respiratory disorder; severe neonatal asphyxia [54,55,56]; surgical condition; PDA requiring treatment [57]; early-onset bacterial infection; death due to infection; use of catecholamines, steroids, and gamma globulin; age at time of the examination; CRP levels; WBC counts; IgM levels; DOT per 1000 patient days; and number of patients receiving an antibiotic were performed.

GDM is defined by glucose intolerance with onset or first recognition during pregnancy. The diagnosis of GDM is established through an oral glucose tolerance test (OGTT), which meets one or more of the following criteria: fasting blood glucose ≥ 92 mg/dL, 1 h value ≥ 180 mg/dL, or 2 h value ≥ 153 mg/dL in the 75 g OGTT [47,48,49]. HDP is defined by blood pressure readings equal to or exceeding 140/90 mmHg during pregnancy [49,50]. Clinical chorioamnionitis was diagnosed when (1) the mother had a fever of 38.0 °C or higher, and one or more of the following four conditions were present: maternal tachycardia (≥100/min), uterine tenderness, malodorous vaginal discharge/amniotic fluid, or maternal WBC count ≥15,000/µL; and (2) when all four conditions were present and other diseases were ruled out, even if the maternal body temperature was lower than 38.0 °C [51,52]. PROM was defined as water breaking before the start of labor [53]. Respiratory disorder was defined as cases with apneic attacks, cases requiring oxygenation, high-flow nasal cannula, continuous positive airway pressure, and artificial mechanical ventilation. Severe neonatal asphyxia was defined as a 1 min Apgar score of 3 or less [54,55,56]. Surgical condition was defined as cases that underwent any surgeries by 6 days of age. PDA requiring treatment was defined as cases requiring surgical ligation or drug therapy, such as use of indomethacin [57]. Early-onset bacterial infection was diagnosed as described (Section 4.3). In the Post-Group, PCT values were measured in all neonates admitted in our NICU. In cases diagnosed with early-onset bacterial infection, the pathogens detected, specimens with positive cultures, and diagnoses were extracted.

#### 4.5.2. Calculation of the DOT

The DOT in one patient was calculated as (total number of antibiotics administered) × 1/(number of antibiotics administered per day), up to 6 days after birth. The DOT per 1000 patient days was calculated by the total DOT of all patients × 1/(number patients × 7 days) × 1000 [58]. The DOT per 1000 patient days was compared between the Pre- and Post-Groups. In cases where more than one type of antibiotic was administered, the DOT was calculated based on the antibiotic with the longest duration of use. Moreover, the DOT for each antibiotic prescribed was compared. The number of patients who used an antibiotic for over 4 days within one week after birth was compared between the Pre- and Post-Groups.

#### 4.5.3. Adjustment for Background Factors

PS was calculated to adjust for bias in the Pre-Group’s and the Post-Group’s background factors. The Pre-Group’s and Post-Group’s frequencies obtained from the PS were compared by creating a histogram. DOT was examined by multiple regression analysis with PS as a covariate. Similarly, we examined the presence or absence of antibiotic use by logistic regression analysis with PS as a covariate.

#### 4.5.4. Statistical Analyses

Fischer’s exact test, Pearson’s *x*^2^ test, or the Mann–Whitney *U* test was used to compare the datasets of the two groups, as appropriate [59]. All statistical analyses were performed using JMP version 14 (SAS Institute Inc., Tokyo, Japan). A two-sided *p*-value of <0.05 was considered statistically significant.

## 5. Conclusions

In NICUs, an introduction of a new method using the 95th percentile cutoff value of the PCT value reference curve can reduce the frequency of prescription and duration of antibiotic use without increasing the incidence of early-onset bacterial infection cases.

## Figures and Tables

**Table 1:** Distribution of maternal and neonatal clinical backgrounds.

	Pre-Group *n* = 737	Post-Group *n* = 686	*p*-Value
A. Maternal backgrounds			
Gestational diabetes	73 (10)	83 (12)	0.19 *
Hypertensive disorder of pregnancy	78 (11)	78 (11)	0.63 *
GBS colonization	99 (13)	98 (14)	0.64 *
Clinical chorioamnionitis	23 (3.1)	12 (1.8)	0.10 *
PROM	152 (21)	75 (11)	<0.01 *
Delivery mode			0.06 *
Vaginal delivery	322 (44)	265 (39)	
Cesarean section	415 (56)	421 (61)	
Precipitate delivery	5 (0.68)	4 (0.58)	1.0 **
Intrapartum antibiotic therapy	483 (66)	474 (69)	0.15 *
B. Neonatal backgrounds			
GA at birth, weeks	37 (22–42)	37 (22–42)	0.49 ***
GA ≥ 37 weeks	421 (57)	366 (53)	0.15 *
28 ≤ GA < 37 weeks	286 (39)	293 (43)	
GA < 28 weeks	30 (4.1)	27 (4.0)	
BW, g	2504 (356–4315)	2448 (278–4446)	0.21 ***
BW ≥ 2500 g	372 (50)	318 (46)	0.12 *
1500 ≤ BW < 2500 g	279 (38)	293 (43)	
1000 ≤ BW < 1500 g	50 (6.8)	42 (6.1)	
BW < 1000	36 (5.0)	33 (4.8)	
Apgar score			
at 1 min	8 (0–10)	8 (0–10)	0.87 ***
at 5 min	9 (0–10)	9 (1–10)	0.07 ***
Neonatal transport	229 (31)	161 (23)	<0.01 *
Respiratory disorder	456 (62)	425 (62)	0.97 *
Severe neonatal asphyxia	90 (12)	63 (9.2)	0.11 *
Surgical condition	20 (2.7)	7 (1.0)	0.02 *
Patent ductus arteriosus that required indomethacin or surgical treatment	30 (4.1)	34 (5.0)	0.42 *
Early-onset bacterial infection	15 (2.0)	14 (2.0)	0.99 *
Death	0 (0)	0 (0)	-
Death due to infection	0 (0)	0 (0)	-
Use of catecholamine	62 (8.4)	75 (11)	0.10 *
Use of steroid	11 (1.5)	11 (1.6)	0.87 *
Use of gamma globulin	34 (4.6)	28 (4.1)	0.62 *
Age at measurement of biomarkers, days	0 (0–3)	0 (0–3)	0.01 ***
PCT, ng/mL	- (-)	0.17 (0.02–100)	-
CRP, mg/dL	0.1 (0.1–10.64)	0.1 (0.1–10.7)	0.01 ***
WBC, /µL	13,600 (1500–53,700)	13,100 (2200–62,600)	0.12 ***
IgM, mg/dL	8 (2–220)	8 (2–253)	0.99 ***

Values are presented as the median (range) or number (percentage). * Data were compared using Pearson’s chi-square test. ** Data were compared using Fischer’s exact test. *** Data were compared using Mann–Whiney *U* test. BW, body weight; GA, gestational age; CRP, C-reactive protein; GBS, group B *Streptococcus*; IgM, immunoglobulin M; PROM, premature rupture of membranes; PCT, procalcitonin; WBC, white blood cell.

**Table 2:** The details of early-onset bacterial infection in the Pre-Group.

No.	Detected Pathogens	Culture-Positive Samples	Diagnosis	Delivery Mode	Gestational Age at Birth
1	*Escherichia coli,* *α-Streptococcus*	Gastric aspiration	Clinical sepsis	Vaginal delivery	40 weeks
2	*Streptococcus agalactiae*	Blood, nasopharyngeal swab, and stool	Culture-proven sepsis	Vaginal delivery	39 weeks
3	*Streptococcus agalactiae*	Nasopharyngeal swab and gastric aspiration	Clinical sepsis	Vaginal delivery	39 weeks
4	*Streptococcus agalactiae*	Blood, nasopharyngeal swab, and stool	Culture-proven sepsis	Cesarean section	38 weeks
5	*Staphylococcus aureus*	Nasopharyngeal swab and stool	Clinical sepsis	Vaginal delivery	37 weeks
6	*Streptococcus agalactiae*	Stool	Clinical sepsis	Vaginal delivery	39 weeks
7	*Enterococcus faecalis*	Gastric aspiration	Clinical sepsis	Cesarean section	41 weeks
8	*Streptococcus agalactiae*	Stool	Clinical sepsis	Vaginal delivery	40 weeks
9	*Streptococcus agalactiae*	Gastric aspiration	Clinical sepsis	Cesarean section	39 weeks
10	*Streptococcus equisimilis*	Blood, nasopharyngeal swab, and stool	Culture-proven sepsis	Vaginal delivery	40 weeks
11	*Staphylococcus aureus*	Nasopharyngeal swab and stool	Clinical sepsis	Cesarean section	40 weeks
12	*Enterococcus faecalis*	Nasopharyngeal swab, stool, and gastric aspiration	Clinical sepsis	Cesarean section	33 weeks
13	*Staphylococcus aureus*	Nasopharyngeal swab and gastric aspiration	Clinical sepsis	Cesarean section	25 weeks
14	*Escherichia coli*	Nasopharyngeal swab, stool, and gastric aspiration	Clinical sepsis	Vaginal delivery	34 weeks
15	*Streptococcus agalactiae*	Urine	Clinical sepsis	Vaginal delivery	33 weeks

**Table 3:** The details of early-onset bacterial infection in the Post-Group.

No.	Pathogens Detected	Culture-Positive Samples	Diagnosis	Delivery Mode	Gestational Age at Birth
1	*Escherichia coli*	Blood, nasopharyngeal swab, and gastric aspiration	Culture-proven sepsis	Vaginal delivery	38 weeks
2	*Streptococcus agalactiae*	Nasopharyngeal swab, stool, and gastric aspiration	Clinical sepsis	Cesarean section	41 weeks
3	*Streptococcus agalactiae*	Nasopharyngeal swab, stool, and gastric aspiration	Clinical sepsis	Cesarean section	41 weeks
4	*Streptococcus agalactiae*	Nasopharyngeal swab and stool	Clinical sepsis	Cesarean section	40 weeks
5	*Gardnerella vaginalis*	Stool and gastric aspiration	Clinical sepsis	Cesarean section	27 weeks
6	*Streptococcus pneumoniae*	Nasopharyngeal swab, stool, and gastric aspiration	Clinical sepsis	Cesarean section	33 weeks
7	*Staphylococcus aureus*	Nasopharyngeal swab, stool, and urine	Clinical sepsis	Cesarean section	33 weeks
8	*Escherichia coli*	Nasopharyngeal swab	Clinical sepsis	Cesarean section	25 weeks
9	*Coryneform* Gram-positive rod	Gastric aspiration	Clinical sepsis	Vaginal delivery	35 weeks
10	*Escherichia coli*	Nasopharyngeal swab and urine	Clinical sepsis	Cesarean section	35 weeks
11	*Prevotella bivia*	Gastric aspiration	Clinical sepsis	Cesarean section	22 weeks
12	*Prevotella bivia*	Gastric aspiration	Clinical sepsis	Cesarean section	25 weeks
13	*Streptococcus agalactiae*	Nasopharyngeal swab and stool	Clinical sepsis	Vaginal delivery	22 weeks
14	*Gardnerella vaginalis*	Nasopharyngeal swab, stool, and gastric aspiration	Clinical sepsis	Cesarean section	30 weeks

**Table 4:** Comparison of antibiotic therapies and DOT between the Pre- and Post-Groups.

	Pre-Group *n* = 737	Post-Group *n* = 686	*p*-Value
Antibiotic therapy	280 (38)	79 (12)	<0.01 *
DOT	211.3	82.0	<0.01 **
Ampicillin DOT	203.3	74.5	<0.01 **
Amikacin DOT	133.2	62.8	<0.01 **
Cephalosporin DOT	8.8	8.0	0.88 **
Penicillin DOT	0	1.4	0.30 **
Meropenem DOT	0	0.9	0.30 **
Piperacillin-tazobactam DOT	0	0.5	0.30 **
Vancomycin DOT	0	0	-

* Data are presented as number (percentage) and were compared using Pearson’s chi-square test. ** Data are presented as the total number of DOT and were compared using Mann–Whiney *U* test. DOT indicates the days of antibiotic therapy per 1000 patient days.

**Table 5:** The duration of antibiotic use in neonates who received antibiotics.

	Pre-Group *n* = 280	Post-Group *n* = 79	*p*-Value
1–3 days	84 (30)	3 (3.8)	<0.01
4–7 days	196 (70)	76 (96.2)	

Data are presented as number (percentage) and were compared by using Pearson’s chi-square test.

**Table 6:** Multiple regression analysis.

DOT	Estimate	SE	t-Value	*p*-Value	95% CI	
					Lower	Upper
Intercept	3.1	0.21	14.6	<0.01	2.7	3.5
Intervention, yes	−0.70	0.10	−6.8	<0.01	−0.89	−0.49
Intervention, no (reference)	-	-	-	-	-	-
PS	−3.5	0.44	−8.1	<0.01	−4.4	−2.7

CI, confidence interval; DOT, days of therapy; PS, propensity score; SE, standard error.

**Table 7:** Logistic regression analysis.

Antibiotic Therapy	Odds Ratio	*p*-Value	95% CI	
			Lower	Upper
Intervention, yes	0.26	<0.01	0.20	0.35
Intervention, no (reference)	-	-	-	-
PS	0.0081	<0.01	0.0026	0.025

CI, confidence interval; PS, propensity score.

## Data Availability

The data of this study are available from the corresponding author upon reasonable request.
